# Supplementary material for: Exploring the Regulators of Keratinization: Role of BMP-2 in Oral Mucosa
Source: Cells. 2024 May 9;13(10):807. doi: 10.3390/cells13100807 (PMC11119837; doi:10.3390/cells13100807)
Supplement: Supplementary file 1 [file cells-13-00807-s001.zip › cells-2964801-SI.pdf]

Supplementary Materials

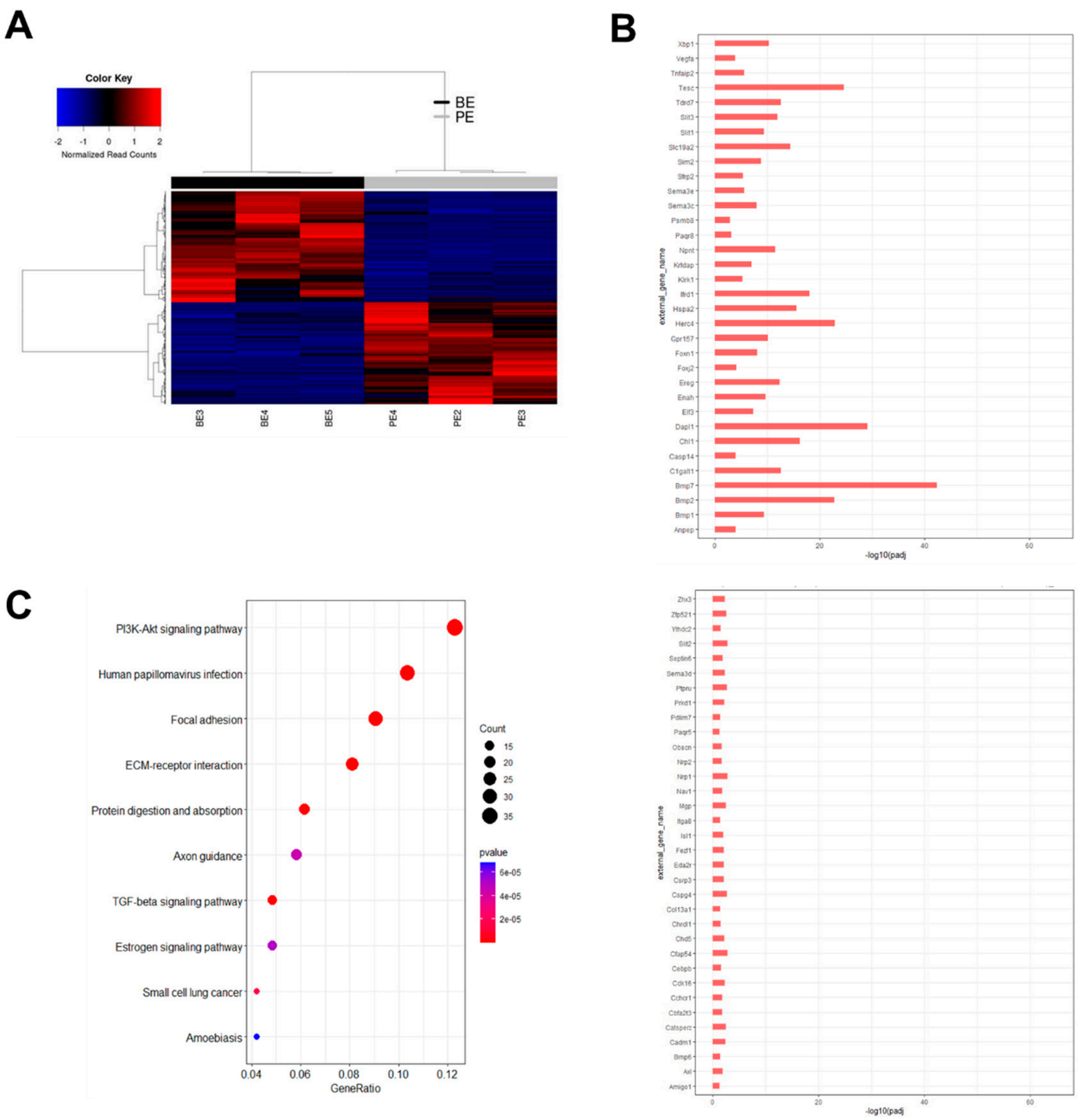

Figure S1. Comparison of the epithelial tissue between keratinized and non-keratinized epithelium.

(**A**) The heatmap shows the comprehensive differences in the gene expression between keratinized and non-keratinized epithelium (*n*=10 mice/group, 3 groups).

(**B**) Sixty-seven differentially expressed genes (DEGs) from epithelial comparison were associated with the Uniprot keyword “Differentiation”.

(**C**) Over Representation Analysis revealed 10 of the most significant KEGG pathways from the upregulated DEGs.

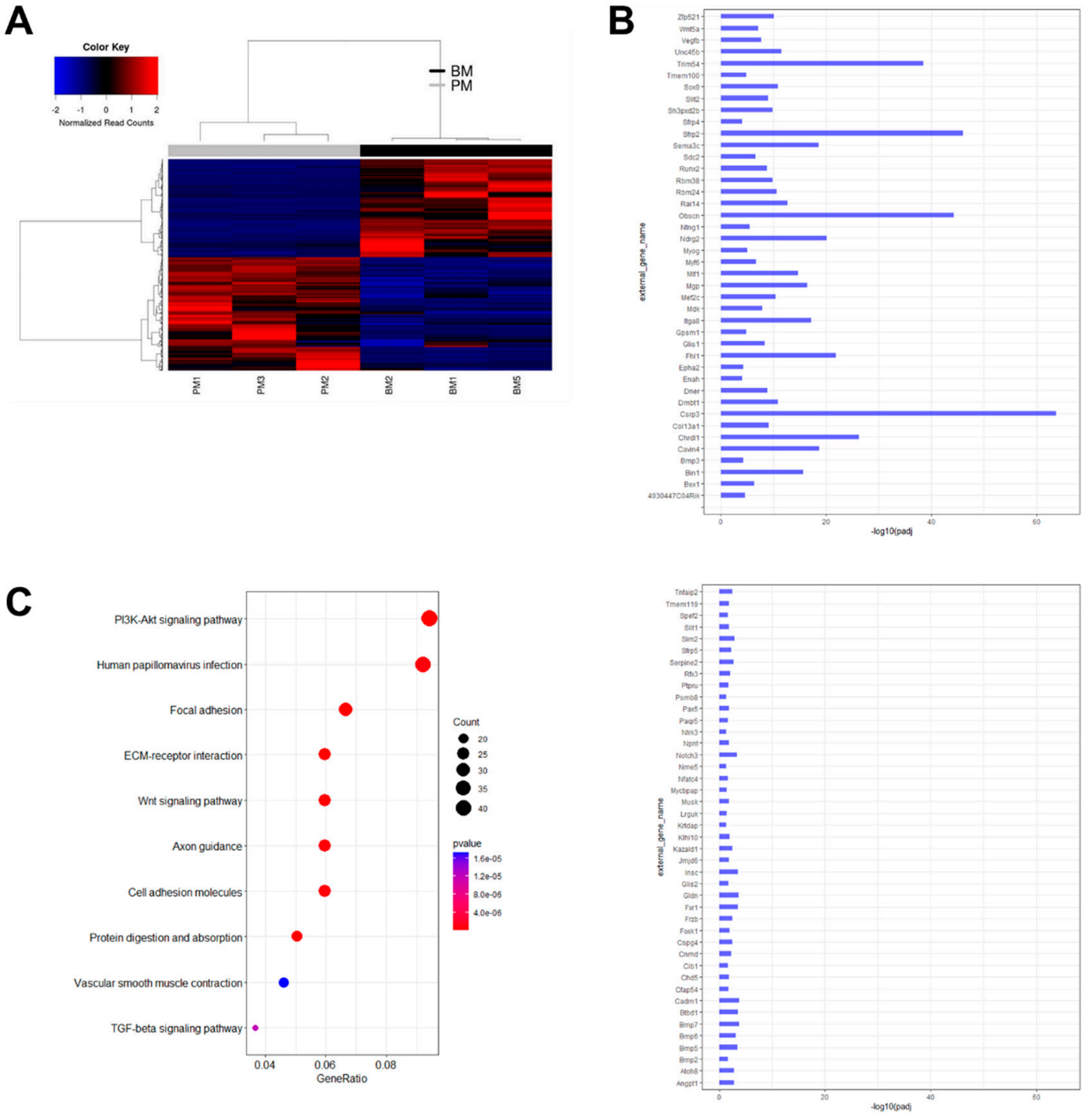

Figure S2. Comparison of the mesenchymal tissue between keratinized and non-keratinized mucosa.

(**A**) The heatmap shows the comprehensive differences in the gene expression between the mesenchyme of keratinized and non-keratinized mucosa (*n*=10 mice/group, 3 groups).

(**B**) Eighty-five differentially expressed genes (DEGs) from mesenchymal comparison were associated with Uniprot keyword “Differentiation”.

(**C**) Over Representation Analysis revealed 10 of the most significant KEGG pathways from the upregulated DEGs.

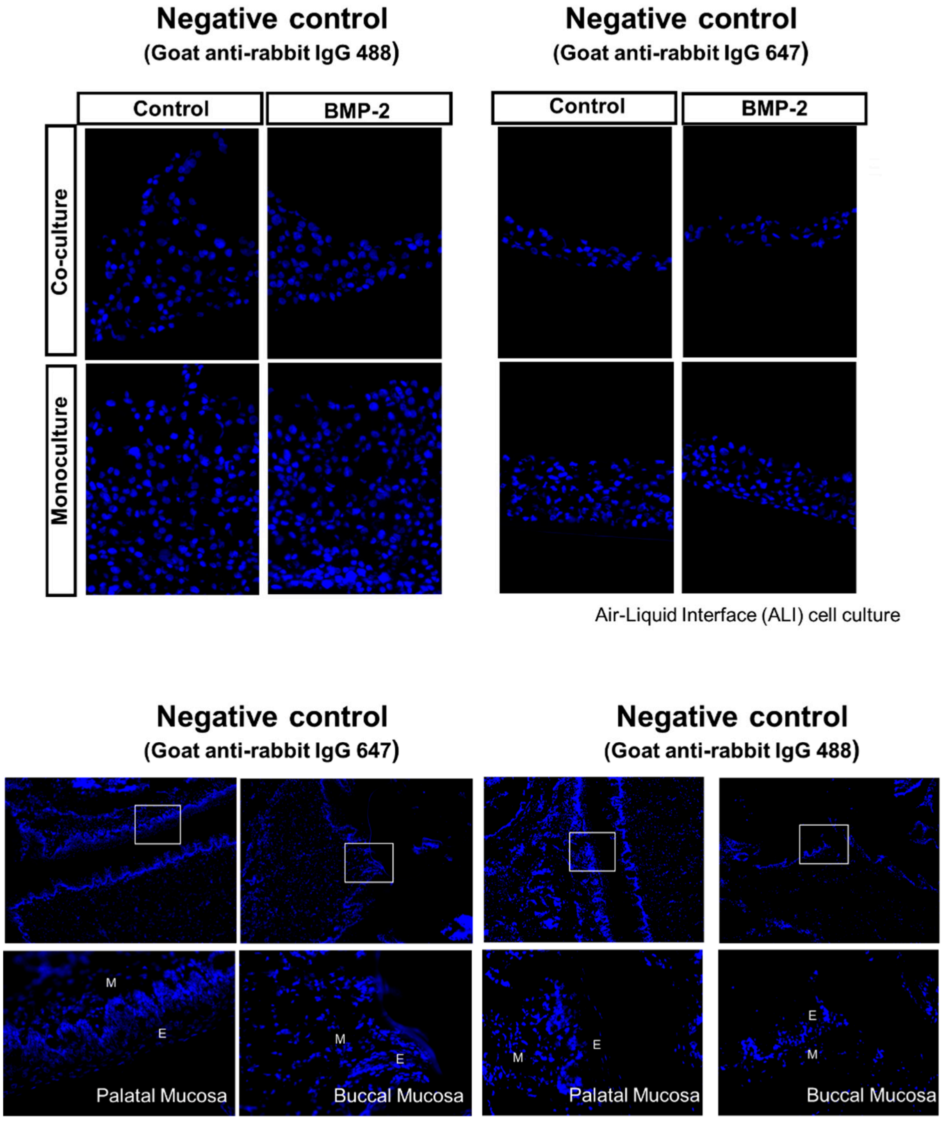

Figure S3. Negative control of immunofluorescence analysis.

The negative control without primary antibody in the Air-Liquid Interface (ALI) cell culture, palatal mucosa, and buccal mucosa. E: Epithelium; M: Mesenchyme.

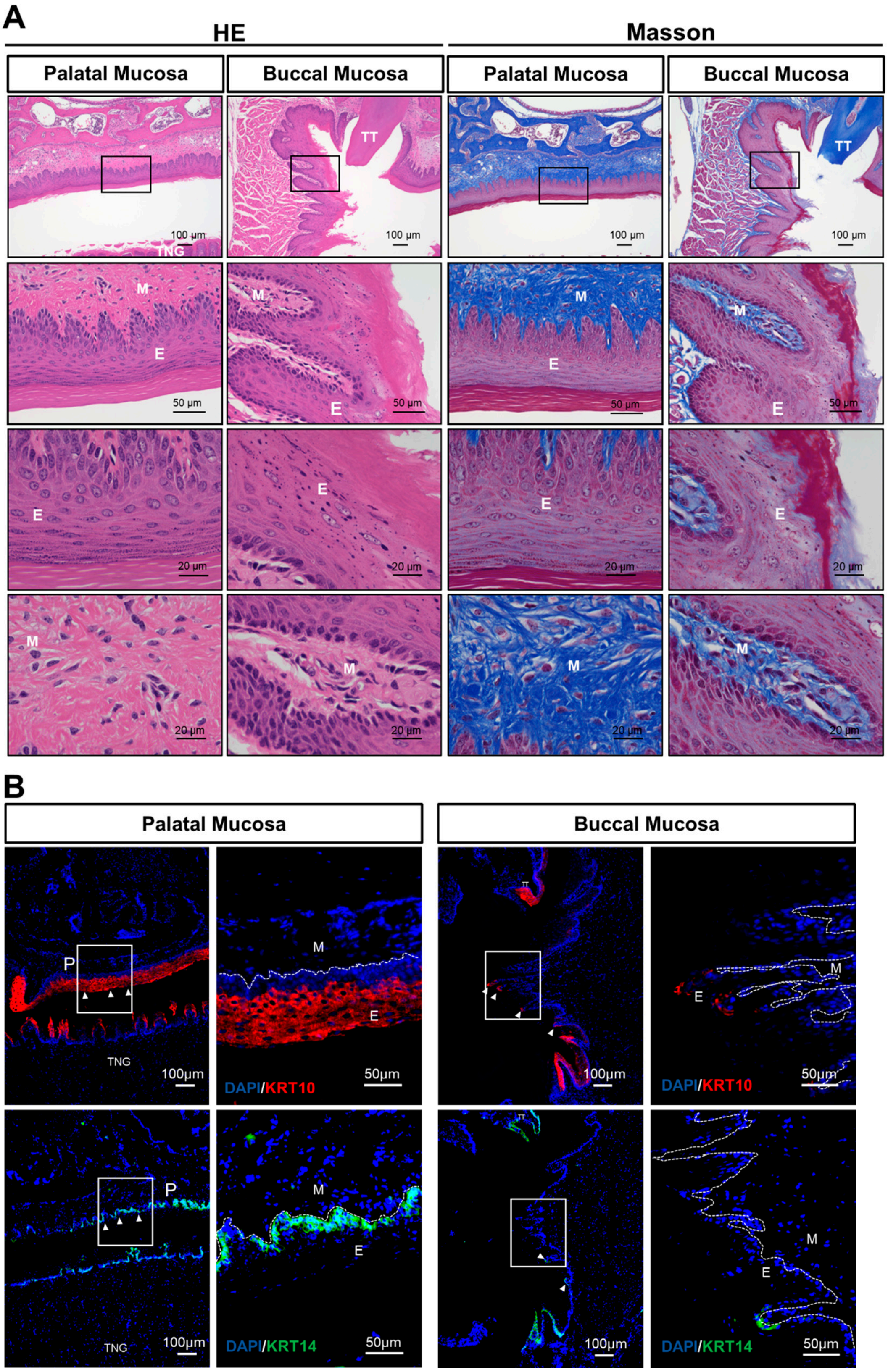

Figure S4. Histological features of palatal and buccal mucosa.

(**A**) HE and Masson's trichrome staining of palatal and buccal mucosa from 8-week-old mice. A sheet-like structure composed of accumulated dead cells was observed in the outermost

layer of both buccal and palatal mucosa. The palatal mucosa presented more flat and tightly packed cells in stratified epithelial layers, while the connective tissue of buccal mucosa was thinner and had less densely organized collagen fibers.
(**B**) Immunofluorescence analysis indicating the distribution of KRT10 and KRT14 in palatal and buccal mucosa. Both proteins were highly expressed in the epithelium of palatal mucosa, but only several scattered signals were detected in buccal mucosa. Arrowheads indicate positive cells for KRT10 and KRT14. E, Epithelium; M, Mesenchyme; TT, Tooth; TNG, Tongue.

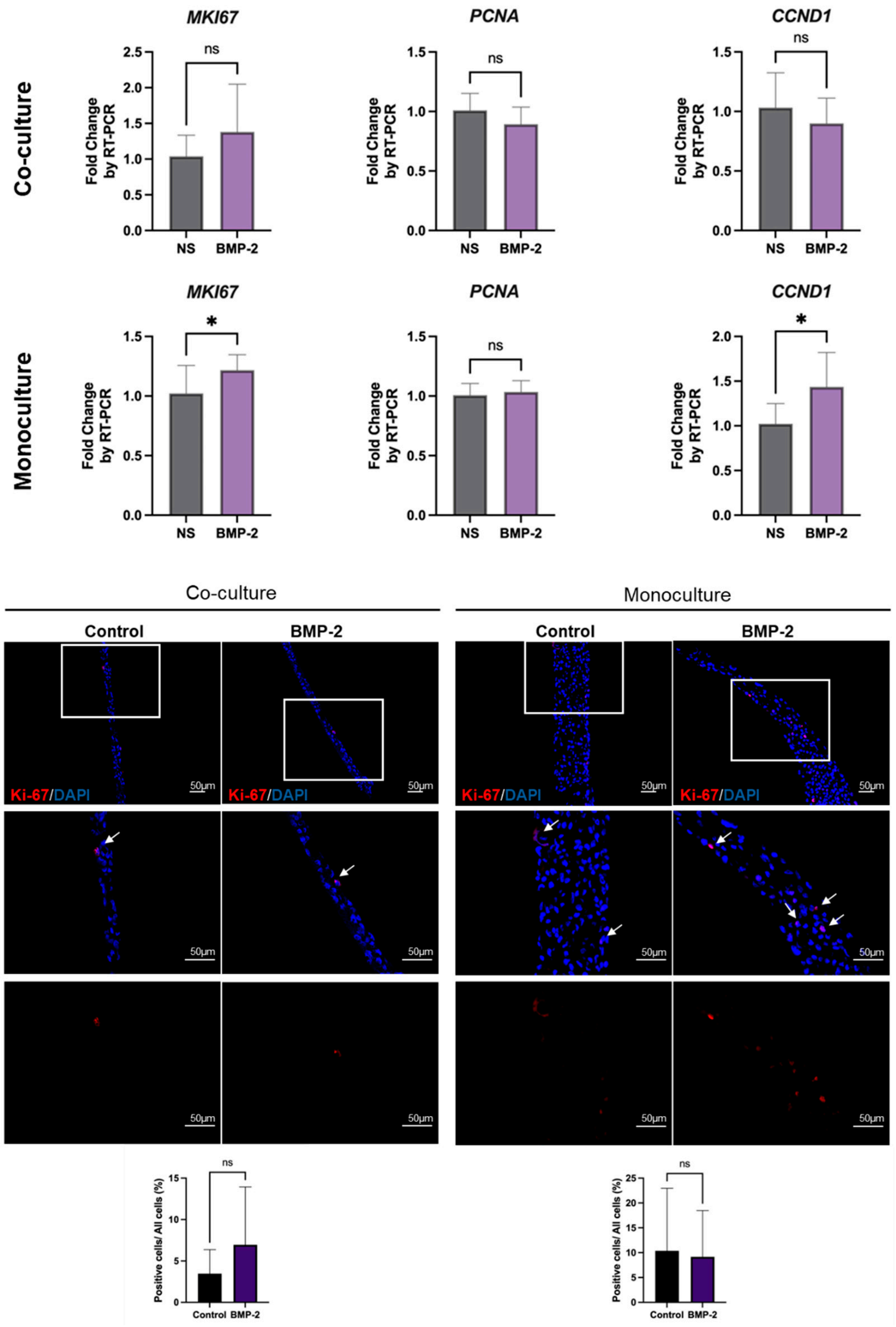

Figure S5. The expression of proliferation markers in Air-Liquid Interface (ALI) cell culture.

Quantification of proliferation genes after treatment with BMP-2 in co-cultured and monocultured ALI by RT-qPCR and immunofluorescence staining analysis. Arrowheads indicate positive cells for Ki67. Scale bar: 50 μm. The graphs show the number of positive cells/total cells (%). All experiments were performed in triplicate (*n*=3). Data are expressed

as mean ± SD. ns=non-significant. * $P \leq 0.05$, ** $P \leq 0.01$, *** $P \leq 0.001$, two-tailed unpaired t test.

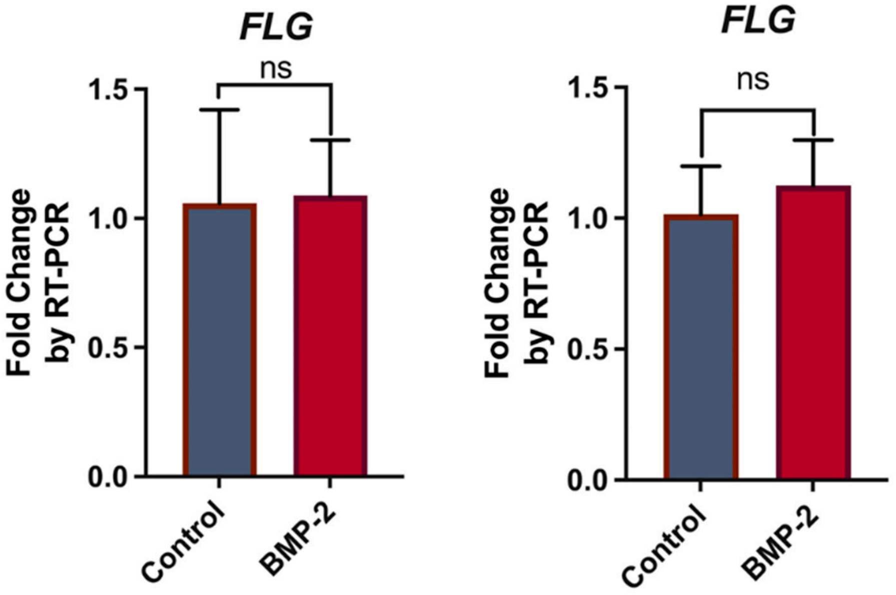

Figure S6. The gene expression of epithelial late differentiation marker in Air-Liquid Interface (ALI) cell culture.

After stimulating by BMP-2, the gene expression of FLG in both the co-cultured and monocultured epithelial cells (TR146) was measured by RT-qPCR analysis. All experiments were performed in triplicate (*n*=3). Data are expressed as mean ± SD. Significance values were calculated with two-tailed unpaired t test. ns=non-significant.

Table S1. The design of PCR primers.

Target gene	Type	GeneBank registration number	Primer set
S29	human	BC032813	5′-TCTCGCTCTTGTCGTGTCTGTTC-3′(S)
			5′-ACACTGGCGGCACATATTGAGG-3′(AS)
KRT10	human	NM_000421	5′-ACACCGCACAGAACCACCACTC-3′(S)
			5′-GGCAGGCACAGGTCTTGATGAAC-3′(AS)
KRT14	human	BC042437	5′-ACAGATCCCACTGGAAGAT-3′(S)
			5′-AGATAATGAAGCTGTATTGATTGC-3′(AS)
KRT15	human	BT007261	5′-TGCTGCTTGACATAAAGACA-3′(S)
			5′-CTACCACCACCTCCTGAA-3′(AS)
KRT16	human	AF061812	5′-TGAGATGGAGCAGCAGAG-3′(S)
			5′-GCGGGAAGAATAGGATTGG-3′(AS)
IVL	human	BC046391	5′-CCTCAGATCGTCTCATACAAG-3′(S)
			5′-ACAGAGTCAAGTTCACAGATG-3′(AS)
FLG	human	NM_002016	5′-AGACTCTAGTACCGCTAAGG-3′(S)
			5′-CGTGACTGTATTCCTGAGTG-3′(AS)
LTBP1	human	NM_206943	5′-TCTTACCAGTGCGTTCCCTG-3′(S)
			5′-TCTACAGTGCTTGTGGTCCG-3’ (AS)
SERPINE1	human	NM_000602	5′-TGACTGGGTGAAGACACACAC-3′(S)
			5′-GAGTCGGGGAAGGGAGTCTT-3’ (AS)
IGF2	human	NM_000612	5′-GACACCCTCCAGTTCGTCTG-3′(S)
			5′-GCGGAAACAGCACTCCTCAA-3’ (AS)
LOX	human	NM_002317	5′-GGATTGAGTCCTGGCTGTTATGA-3′(S)
			5′-GTGTAGCGAATGTCACAGCG-3’ (AS)
IL24	human	NM_006850	5’-ATTCTACAAGCTCTGAATGTCT-3’ (S)
			5’-CGTGAAGTGTCCAGTGAAC-3’ (AS)
MMP1	human	BT006874	5’-AAGCGTGTGACAGTAAGC-3’ (S)
			5’-CGGGTAGAAGGGATTTGTG-3’ (AS)
ID1	human	NM_002165	5′-TGCCTAAGGAGCCTGGAAAAAG-3′(S)
			5′-ATGCCGCCTGTGAAAACGAG-3’ (AS)
Mki67	human	NM_002417	CACTCCACCTGTCCTGAAGA
			GCAGGTTGCCACTCTTTCTC
PCNA	human	NM_182649	ACCAGGAGAAAGTTTCAGACTATG
			GTGCAAATTCACCAGAAGGCA
CCND1	human	NM_053056	CACTCCTACGATACGCTACTA
			GCATCTCATAAACAGGTCACT
